# Service-Learning for Cardiopulmonary Resuscitation Training in Nursing Students: A Qualitative Study

**DOI:** 10.3390/nursrep16060186

**Published:** 2026-05-28

**Authors:** Verónica V. Márquez-Hernández, Jessica García-González, Miguel Company-Morales, Diego Ruiz-Salvador, José Miguel Garrido-Molina, Alba García-Viola, María Margarita Lirola-González, Mᵃ Carmen Rodríguez-García

**Affiliations:** 1 Department of Nursing, Physiotherapy and Medicine, Universidad de Almería, 04120 Almería, Spain; vmh380@ual.es (V.V.M.-H.); mcm107@ual.es (M.C.-M.); rsd375@ual.es (D.R.-S.); mrg451@ual.es (M.C.R.-G.); 2 Seron Primary Care Center, Northern Almería Integrated Healthcare Area, 04600 Huercal-Overa, Spain; 3 Hospital Universitario de Poniente, 04700 Almería, Spain; mariam.lirola.sspa@juntadeandalucia.es; 4 Hospital HLA Mediterráneo, 04007 Almería, Spain; josemiguel.garrido.sspa@juntadeandalucia.es; 5 Distrito Sanitario Almería, 04007 Almería, Spain; alba.garcia.sspa@juntadeandalucia.es

**Keywords:** cardiopulmonary resuscitation, qualitative research, nursing, nursing students, service learning

## Abstract

**Background/Objective:** Service-learning is an educational methodology that has demonstrated benefits and effectiveness in nursing education, generating positive outcomes for both students and the community. However, its application in cardiopulmonary resuscitation (CPR) training remains underexplored. This study explored nursing students’ perceptions of CPR training through service-learning, focusing on dimensions of learning, emotional experience, professional identity, and perceived difficulties in implementation. **Methods:** A descriptive qualitative study was conducted with 30 nursing students selected through purposive sampling. Data were collected via semi-structured interviews, which were audio-recorded, transcribed verbatim, and thematically analyzed using ATLAS.ti. **Results:** Four main themes emerged: (1) meaningful learning and consolidation of competencies; (2) emotional impact associated with teaching CPR; (3) increased social awareness and strengthening of professional identity; and (4) perceived difficulties and barriers in implementing service-learning. **Conclusions:** The results suggest that nursing students perceived service-learning in CPR training as a meaningful learning experience, associated with greater self-confidence, greater emotional engagement, and greater social awareness, despite the perceived barriers.

## 1. Introduction

In Europe, it is estimated that approximately 275,000 people experience an out-of-hospital cardiac arrest (OHCA) each year, of whom 29,000 survive when bystanders initiate early CPR [1]. In Spain, an estimated 52,300 cardiac arrests occur annually, with 30,000 taking place in community settings and 22,300 in hospital settings [2]. The immediate initiation of high-quality CPR, through chest compressions, defibrillation, or both, is one of the most important predictive factors for improving outcomes following cardiac arrest [3]. Bystander-initiated CPR significantly improves survival rates in out-of-hospital cardiac arrests and represents a key target for health professionals to focus their efforts on increasing CPR uptake rates [4].

Knowledge of CPR procedures among nursing students is considered an essential training need that should be grounded in up-to-date evidence [5]. This competency is included in specific courses within the various existing university curricula. Effective CPR training requires the optimal use of educational strategies [6]. One educational methodology that has demonstrated benefits and effectiveness in nursing education is service-learning. Service-learning is part of innovative teaching approaches that emphasize the active role of students and address real-world issues that are meaningfully connected to the educational context [7]. According to Furco [8], service-learning aims to engage students in activities that combine community service with academic learning, while recognizing that such programs are typically grounded in curricular educational content. The pedagogy of service-learning is based on three core components: experiential learning, reciprocal learning, and reflection [9].

Service-learning has been implemented across numerous health sciences programs, including nursing, pharmacy, physiotherapy, and medicine [10,11]. In this regard, service-learning not only enhances educational skills, such as the reinforcement of concepts and techniques, but also promotes educational innovation by providing a transversal and complementary approach [12]. Currently, service-learning is widely incorporated into nursing curricula for undergraduate students, graduate students, registered nurses, and advanced practice nurses [13]. Recent studies indicate that service-learning programs involving the active participation of nursing students generate benefits for both the students and the community [14]. Specifically, these interventions receive positive evaluations from participating students. Moreover, the interventions implemented are diverse and require motivation and creativity for their execution [15]. These activities provide an opportunity for nursing schools to achieve greater visibility within society. Partnerships at the community level benefit students, nursing faculty, and the recipients of the activities [16,17]. Specifically, this methodology can be particularly advantageous in resource-limited settings or in communities composed of underserved populations [14].

Within formal education, opportunities to develop competencies related to health education outside the hospital setting are limited. Given the importance of bystander intervention in witnessed cardiac arrests, the potential to promote education and social responsibility among participants, and the demand for training activities on this topic, it is particularly relevant to conduct research that advances public health education, empowers participants, and may contribute to reducing disparities in community access to knowledge. This aligns with the educational recommendations on resuscitation outlined in the most recent international guidelines [18].

Despite the well-established benefits of service-learning in nursing education and the recognized importance of bystander CPR training, an important gap remains in the literature regarding its specific application to CPR education. While previous research has examined service-learning in diverse nursing contexts such as community health, geriatrics, and chronic disease management [13,15], its use in CPR training has not been systematically explored, particularly in terms of how nursing students experience, interpret, and integrate resuscitation skills acquired through community-based teaching. This represents a relevant educational gap, as service-learning could potentially contribute not only to improving CPR competence in nursing students, but also to strengthening their role as multipliers of bystander CPR training in the community, thereby supporting public health strategies aimed at improving survival after out-of-hospital cardiac arrest [18].From an educational perspective, service-learning has been associated with several advantages in CPR-related training. It promotes experiential and contextualized learning, which may enhance long-term retention compared with traditional didactic approaches [19]. It has also been linked to increased clinical self-efficacy and greater confidence in teaching health-related skills [20,21], as well as the development of transversal competencies such as communication, empathy, and social responsibility [15]. However, the evidence base also highlights important limitations and areas of uncertainty. Service-learning interventions are often heterogeneous in design and intensity, which may affect the consistency of learning outcomes. In addition, logistical demands, variability in community engagement, and the lack of standardized, objective assessment tools for CPR performance remain important challenges. Furthermore, it is still unclear whether perceived improvements in confidence and learning necessarily translate into objectively measured CPR competence or high-quality resuscitation performance in real or simulated settings, indicating a need for more critically balanced research in this area.

Within this context, the objective of the present study was to explore nursing students’ perceptions of CPR training through service-learning, focusing on dimensions of learning, emotional experience, professional identity, and perceived difficulties in implementation.

## 2. Materials and Methods

### 2.1. Design

A descriptive qualitative study was conducted [22]. This methodology allowed for a thematic analysis of nursing students’ perceptions of CPR training through service-learning, following the guidelines of the Consolidated Criteria for Reporting Qualitative Research (COREQ) [23].

### 2.2. Participants

The participants were students enrolled in the Bachelor of Nursing program at the University of Almería (Spain). Intentional sampling was used to select participants. Inclusion criteria were: (a) enrollment in the “Nursing in Emergency Care” course within the Bachelor of Nursing program, and (b) attendance at theoretical–practical training in basic life support. Students repeating the course who had previously participated in the activity were excluded. The appropriate number of informants was determined using the saturation criterion [24]. In this study, saturation was operationally defined as the point at which no new codes or meaning units emerged during successive interviews, despite continued data collection and analysis occurring in parallel. To enhance transparency and replicability, saturation was assessed through an iterative process of concurrent data collection and analysis, with preliminary coding conducted after each interview to monitor the emergence of new themes. Data collection took place between September and November 2025 through individual semi-structured interviews with native Spanish-speaking participants. Data collection continued progressively until thematic saturation was reached [25].

### 2.3. Data Collection

The procedure was conducted in the following phases:

Phase 1: project information: Through various institutional channels, the research team offered the opportunity for CPR training to be provided by students enrolled in the “Nursing in Emergency Care” course of the Bachelor of Nursing program, targeting different educational centers, associations, and/or vulnerable groups. Initial coordination meetings were held with representatives from participating schools, sports centers, and cultural associations to define logistical aspects of the intervention, including session format, duration, participant profiles, and available facilities. Community groups were contacted through official university channels, ensuring alignment with institutional coordination processes.

Phase 2: participant recruitment: Participation was voluntary but regulated through the course syllabus. Students enrolled in the “Nursing in Emergency Care” course were invited to participate. They received information about the project’s objectives, study characteristics, voluntary participation, and the anonymous handling of data. All participants signed written informed consent. It should be explicitly acknowledged that the members of the research team were also the faculty instructors responsible for the “Nursing in Emergency Care” course.

Phase 3: activity planning: Training activities were assigned based on existing demand and the participants involved. The intervention was conducted in a group format. Each student participated in 1 to 3 training activities within the group setting. All students were supported by a tutor from the research team, who supervised the entire intervention process. Training materials (adult mannequins, AED trainers, and CPR informational brochures) were centrally managed by the research team and distributed to students prior to each session. Session scheduling was coordinated with each receiving institution according to calendar availability, ensuring that all activities were conducted within the first semester of the academic year and adapted to the characteristics of each recipient group (schoolchildren, sports center members, and cultural association members). Regarding curriculum design, students first received theoretical instruction in Basic Life Support (BLS) according to the European Resuscitation Council guidelines. This was followed by supervised practical simulation training in the nursing skills laboratory, using CPR manikins and automated external defibrillator (AED) simulators. Preparatory activities also included peer practice sessions, role-playing exercises involving cardiac arrest scenarios, and structured debriefing sessions with faculty tutors. Teaching strategies emphasized hands-on practice with manikins and AED simulators.

Phase 4: Activities were scheduled during the first semester of the academic year as part of the “Nursing in Emergency Care” course and were conducted after students completed their initial academic training. During practical classes, students received the necessary preparation to carry out the interventions. Each community CPR training session lasted approximately 60–90 min and was delivered in groups of 10 to 25 participants depending on the setting.

The instructional methodology combined brief didactic explanations of cardiac arrest recognition and the chain of survival, hands-on demonstration of chest compressions and rescue breathing using mannequins and AED trainers, and supervised practice by community participants. This approach aligned with the core components of service-learning: experiential learning, reciprocal learning, and reflection. Community members actively participated through hands-on practice, guided questions, and completion of a brief satisfaction survey at the end of each session. The level of engagement was high, as all participating groups completed the full training and expressed interest in future educational activities.

It should be noted that no formal standardized assessment of students’ CPR performance (e.g., using feedback manikins or structured observation checklists) was conducted either before or after the community sessions.

**Figure 1:**
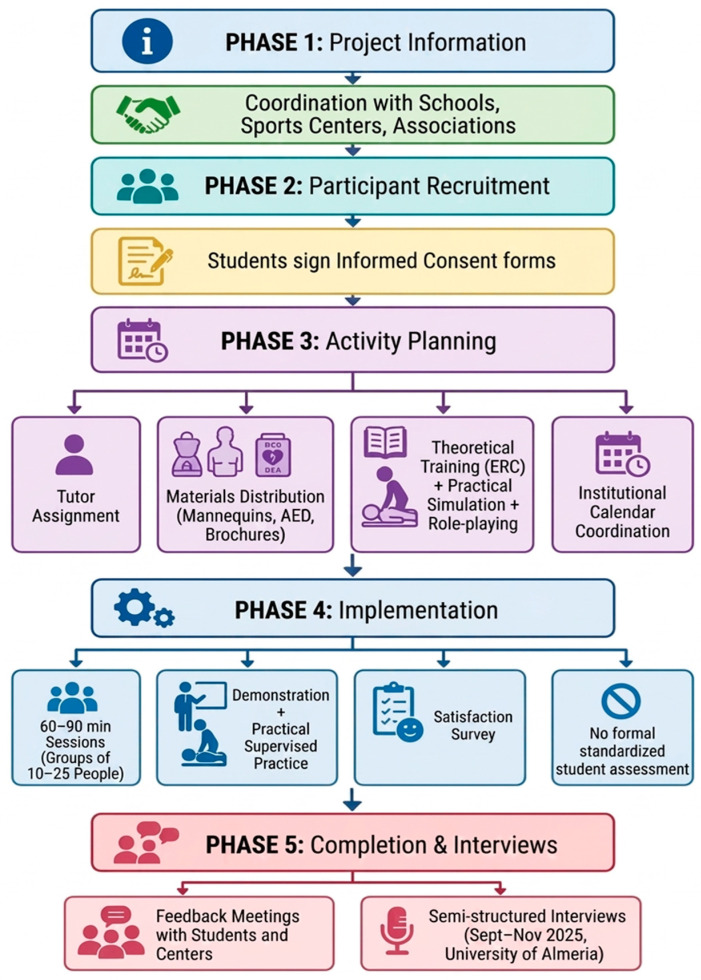
Project phases.

Phase 5: completion of activities and interviews: After all training activities were completed, the research team met with the participating students and centers to collect feedback on satisfaction, suggestions for improvement, and other observations. Data collection took place between September and November 2025 through individual semi-structured interviews with native Spanish-speaking participants. The interviews were conducted in person in a class at the University of Almería (Spain) by a nursing researcher (J.G.-G.) trained in qualitative research, following a thematic guide (Table 1) developed from the existing literature. The guide was piloted by the same researcher with three participants to ensure clarity and appropriateness of the questions. The phases of the project are summarized in Figure 1.

All interviews were audio-recorded with participant consent and lasted approximately 35 min. They were later transcribed verbatim in Spanish and subjected to textual analysis. The interviewer also maintained a reflective journal to record non-verbal cues and contextual observations, which were incorporated into ATLAS.ti25 (ATLAS.ti Scientific Software Development GmbH, Berlin, Germany) for thematic analysis. Prior to the interviews, participants signed written informed consent that included detailed study information. Sociodemographic data were collected via a structured questionnaire. Participants were informed that codes would be used instead of names, and that data would be stored on secure systems with restricted access. Data collection continued progressively until thematic saturation was reached [25]. Saturation was determined through an iterative and inductive process involving simultaneous data collection and preliminary analysis. After each interview, transcripts were coded and compared with previous data to assess whether new codes or meaning units were emerging. Data collection ceased when two consecutive interviews yielded no additional codes or thematic information, indicating that thematic redundancy had been achieved. Transcriptions were anonymized, and participants were given the opportunity to review them before analysis to confirm the accuracy of their contributions.

### 2.4. Data Analysis

The analysis process was conducted using all in-depth interview transcripts, along with the audio recordings and field notes collected by the research team. The recorded interviews were transcribed verbatim and then integrated into a hermeneutic unit, where multiple researchers independently examined them using ATLAS.ti 25 (ATLAS.ti Scientific Software Development GmbH, Berlin, Germany)The thematic analysis followed the six steps described by Kiger and Varpio [26]: (1) familiarization with the data; (2) generation of initial codes; (3) identification of preliminary themes based on coding; (4) development and review of themes; (5) definition and naming of themes; and (6) report production. The transcripts, codes, and organization into themes and subthemes were reviewed collectively by the research team to ensure validity. During this phase, a table was created including themes, subthemes, and units of meaning. Finally, participants provided feedback on the results obtained. Any discrepancies or disagreements arising during coding and theme development were resolved through iterative discussion among the research team until consensus was achieved. In cases where consensus could not be reached initially, transcripts and codes were re-examined collectively to ensure a shared and coherent interpretation of the data. This collaborative process contributed to the rigor and consistency of the thematic analysis.

### 2.5. Rigour

To ensure the rigor of this study, the quality criteria proposed by Guba and Lincoln [27] were applied. These included: (1) Credibility: Data were analyzed independently by the researchers and subsequently triangulated among all team members. Coding and theme development were compared and discussed until consensus was reached. Additionally, two experts with experience in qualitative research reviewed the analysis to enhance the trustworthiness of the findings. (2) Transferability: A detailed description of the method, study setting, participant characteristics, and overall context was provided to allow readers to assess the applicability of the findings to other contexts. (3) Dependability: An external expert with experience in qualitative research verified the analysis to ensure consistency and coherence throughout the analytical process. (4) Confirmability: All researchers independently examined the transcripts to reach consensus on the emerging units of meaning, as well as the themes and subthemes. In addition, reflexivity was ensured through the maintenance of a reflective journal by the interviewer, documenting methodological decisions, contextual observations, and potential sources of bias during data collection and analysis. Participants were also given the option to review and clarify the interpretation of their transcripts.

### 2.6. Ethical Aspects

This study was conducted in accordance with the ethical principles of the Declaration of Helsinki. Approval was obtained from the Ethics and Research Committee of the Department of Nursing, Physiotherapy, and Medicine at the Faculty of Health Sciences (EFM 460.25). All participants were informed about this study’s objectives, methodology, and their rights. In addition, written informed consent was obtained from all participants prior to the start of this study. Participants were also informed of their right to withdraw at any time, as well as the confidential and anonymous handling of their data, in compliance with the European Data Protection Regulation.

## 3. Results

**Figure 2:**
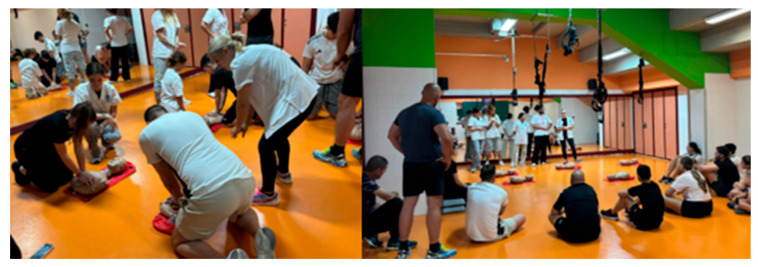
CPR training activity through service-learning at a sports center.

**Figure 3:**
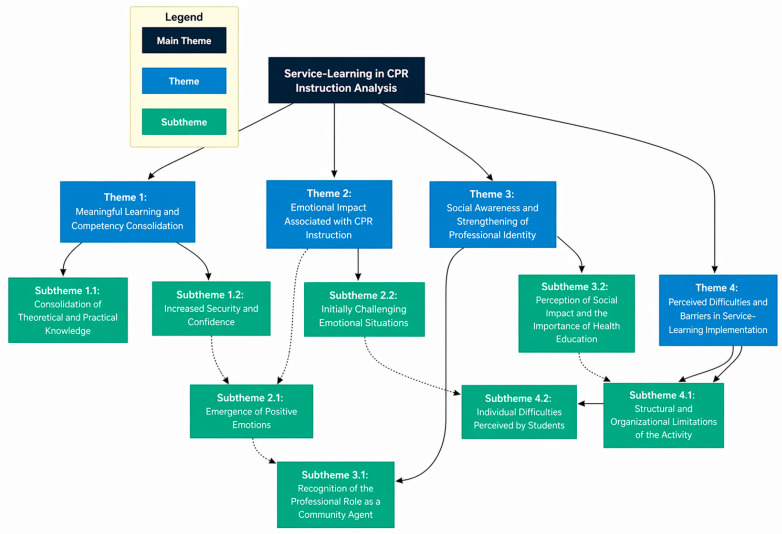
Conceptual map of themes and subthemes.

A total of 30 students participated, of whom 93.33% (*n* = 28) were women and 6.67% (*n* = 2) were men. The mean age was 23.50 years (SD = 7.29). Regarding the location of the activities, 73.33% (*n* = 22) took place in educational centers, 20% (*n* = 6) in sports centers (Figure 2), and 6.67% (*n* = 2) in cultural associations. Detailed data are presented in Table 2. The qualitative analysis identified four main themes and eight subthemes (Figure 3) that describe nursing students’ perceptions of their participation in a CPR training activity through service-learning. The structure of the themes, subthemes, and units of meaning is presented in Table 3.

Theme 1. Meaningful Learning and Competency Consolidation

Participants indicated that the experience of teaching CPR to the community served as a key factor in reinforcing their own learning. The need to explain procedures to others prompted them to review content, consolidate concepts, and improve their understanding of the sequence of actions in a cardiac arrest. Additionally, they reported an increased sense of confidence and ability to intervene in real-life situations.

Subtheme 1.1. Consolidation of Theoretical and Practical Knowledge

The experience with the activity involved not only teaching but also served as a learning mechanism, requiring participants to review both theoretical and practical content, thereby reinforcing their understanding. Service-learning enhances meaningful learning.

*“It helps me consolidate my knowledge, review it, and reinforce it. When you teach what you know, the learning is doubled”*

*(E30)*

Moreover, the activity provided the opportunity to apply CPR procedures in practice, reinforcing the sequence of actions.

*“It is a way of teaching that combines theory with practice and forces you to understand a topic or procedure, since afterwards, you are the one who has to explain it to others”*

*(E19).*

Subtheme 1.2. Increased security and confidence

Students reported an increase in confidence in taking action and in their perception of safety when facing life-threatening situations. This improvement was associated with active practice of CPR and with teaching the procedures to others in a controlled environment.

*“I feel better prepared to handle real-life CPR situations because the activity allowed me to practice the maneuvers multiple times and to teach others, which reinforced my confidence and sense of safety. Additionally, addressing students’ doubts and errors made me reflect on each step and how to act correctly, which helps me remain more aware and prepared for an actual emergency”*

*(E12)*

Theme 2. Emotional Impact associated with CPR Instruction

The emotional dimension played a significant role in the participants’ experience. Most reported predominantly positive emotions, such as satisfaction, motivation, and gratitude, along with the opportunity to convey useful knowledge to the general population. The activity was frequently described as enriching and rewarding. However, some participants reported feeling nervous initially, particularly at the beginning of the sessions.

Subtheme 2.1. Emergence of Positive Emotions

Participants expressed a high level of satisfaction with the activity. The opportunity to share both theoretical and practical knowledge contributed to a positive appraisal of the experience.

*“I truly gained a valuable learning experience and feel very satisfied for contributing my small part to help future individuals perform proper CPR”*

*(E23)*

The experience was described as enriching, not only from an academic perspective but also on a personal level. Additionally, it served as a source of motivation, highlighting the direct contact with the recipients and the feedback received during the sessions.

*“Motivation and enthusiasm for teaching, along with a sense of responsibility and nervousness about wanting to do it well. I also felt satisfaction and pride in seeing the students learn and take an interest in CPR, which made the experience highly rewarding and enriching”*

*(E12)*

*“I believe it was a highly enriching way to learn Basic Life Support and to experience a less familiar aspect of our profession, such as teaching or community service”*

*(E20)*

Subtheme 2.2. Initially Challenging Emotional Situations

Prior to the activity, some participants reported experiencing emotions such as nervousness, insecurity, and tension. These emotions were associated with facing a new experience, perceived responsibility, and direct interaction with the community. However, these feelings gradually diminished as the session progressed and confidence in their skills increased.

*“At the beginning of the session, I felt a bit nervous, but as it progressed, I gained confidence and a sense of control”*

*(E21)*

Theme 3. Social Awareness and Strengthening of Professional Identity

The activity promoted reflection on the social impact of nursing within the community and the importance of training the general population in basic CPR skills. Participation in the project helped reinforce professional vocation and foster a closer connection with the community through the nursing role.

Subtheme 3.1. Recognition of the Professional Role as a Community Agent

Participants emphasized the role of the nursing professional in the community, reinforcing professional vocation by fostering greater commitment to society and broadening their professional perspective, thereby promoting a deeper understanding of the nursing role.

*“I believe this experience influenced me as a future nursing professional, as it allowed me to broaden my perspective on the profession and enhance my practical and communication skills”*

*(E3)*

The activity facilitated connection with the future professional role by providing concrete experiences in which students could apply nursing competencies. In this way, not only was learning consolidated, but professional identity and the impact that nursing can have on the community were also strengthened.

*“By participating with the community during the Service-Learning activity, I felt useful and motivated. It was a meaningful and rewarding experience because I could share knowledge that can save lives and observe the students’ genuine interest. Additionally, it made me feel more connected to my future role as a nurse and more aware of the impact that health education can have”*

*(E13)*

Subtheme 3.2. Perception of Social Impact and the Importance of Health Education

The experience of teaching CPR to the community allowed students to reflect on the social impact of nursing and the relevance of health education as a tool for health promotion. The activity helped consolidate knowledge and increased confidence in the practice of clinical skills. It also facilitated the development of transversal competencies such as empathy and teamwork.

*“I learned that teaching others reinforces my own knowledge and helps me gain confidence in performing CPR. I also understood the importance of health education and the impact we can have on the community, no matter how small. Additionally, the experience allowed me to develop communication, empathy, and teamwork skills, which will be essential in my future practice”*

*(E14)*

Theme 4. Perceived Difficulties and Barriers in the Implementation of Service-learning

Participants identified various challenges. Some were related to organizational aspects, such as time management, activity coordination, and handling large groups. Others stemmed from personal factors, such as embarrassment when speaking in public or fear of making mistakes during explanations. However, these challenges were perceived as opportunities for learning and future improvement.

Subtheme 4.1. Structural and Organizational Limitations of the Activity

Various challenges associated with the implementation of the activity were identified. Among the main barriers were the limited time available to prepare and conduct the sessions, as well as managing large groups, which posed a challenge both for demonstrating techniques and for ensuring active participation of all attendees. Finally, coordinating the activity required prior planning, organization of materials, and management of spaces. Nevertheless, participants highlighted that the experience allowed them to identify areas for improvement for future iterations.

*“During the implementation of the Service-Learning (S-L) project, I faced challenges such as initial insecurity when teaching CPR, time and resource limitations, and the need to coordinate the team to ensure the activity was conducted in an organized manner”*

*(E4)*

Subtheme 4.2. Individual Difficulties Perceived by Students

Some students identified personal challenges during the activity. The most frequent included embarrassment when presenting in front of others and fear of making mistakes while teaching CPR techniques. They also noted that progressive practice and interaction with the community helped reduce these initial emotions and contributed to increased confidence in performing the activity.

*“The activity helped me overcome the fear and embarrassment of presenting and speaking openly”*

*(E15)*

## 4. Discussion

The aim of this study was to explore nursing students’ perceptions of CPR training through service-learning, focusing on dimensions of learning, emotional experience, professional identity, and perceived difficulties in implementation. Service-learning is a tool that integrates experiential learning, reciprocal learning, and reflection, allowing students to engage directly in community contexts and develop key healthcare competencies.

The findings of this study show that nursing students’ participation in CPR training through Service-learning promoted meaningful learning and the consolidation of both theoretical and practical competencies. Studies in the same vein highlight that service-learning activities allow nursing students to apply theory in real contexts, actively and contextually consolidating knowledge, which fosters meaningful learning beyond the traditional classroom [19]. Additionally, service-learning facilitates competency development [20], integration of knowledge, and understanding of meaningful roles, all of which are key aspects of meaningful learning [28].

In this study, the reported improvement in competencies is based exclusively on participants’ self-perceived learning rather than on objective clinical performance measures, which should be considered when interpreting the results. Evidence from studies employing objective assessment methods—such as Objective Structured Clinical Examinations (OSCEs), standardized performance checklists, and real-time feedback systems using simulation mannequins—consistently demonstrates a discrepancy between perceived competence and actual technical performance. For example, a mixed-methods OSCE study involving 250 nursing students reported that 68% failed to achieve guideline-recommended chest compression depth, 45% did not maintain appropriate compression rates, and only 18% correctly applied automated external defibrillator pads, despite participants reporting high levels of confidence and perceived learning [29]. Likewise, International resuscitation guidelines recommend the implementation of feedback systems during both training and clinical practice, as evidence indicates that the absence of feedback is associated with a progressive decline in CPR skill quality over time. The use of objective and continuous feedback helps maintain adequate levels of competence and reduces skill decay following initial training [6]. The subjective measures reported in this study should be complemented with objective performance-based assessments to more accurately evaluate CPR competence, as self-reported confidence alone may not reliably reflect actual resuscitation skills.

Furthermore, students also reported a significant increase in perceived confidence and self-assurance, consistent with research showing that service-learning effectively enhances participants’ self-efficacy, confidence, and cultural competency development [21], and that experiential learning in real situations contributes to the development of clinical self-efficacy [20]. Findings from previous studies support a positive relationship between self-efficacy and professional identity among nursing students, suggesting that higher levels of self-efficacy may contribute to stronger professional commitment and improved performance over time [30,31]. Moreover, service-learning provides a broader perspective on healthcare and fosters a greater sense of agency and self-confidence [32]. These findings are consistent with research in experiential learning, where repeated practice and application in simulated or real situations contribute to the development of clinical self-efficacy and increased perceived confidence [20].

Participants also highlighted the emotional impact of the activity. Most described positive emotions such as satisfaction, motivation, and gratitude when observing the effect of their actions on the community. Similarly, García-Garcés et al. [33] reported that nursing students experienced very high levels of satisfaction with the service-learning methodology in the course. Additionally, Guerra-Marmolejo et al. [19] emphasize emotional aspects such as motivation and engagement. Previous studies have already demonstrated the significant emotional impact of the service-learning methodology [34]. These emotions appear to be linked to the transformative nature of service-learning, allowing learners to connect learning with personal values and thus reinforcing the affective dimension of the educational process [15]. However, students also reported negative emotions prior to the activity, such as nervousness and insecurity associated with direct contact with the community, which may be attributed to the intrinsic nature of service-learning that connects theory and practice in real contexts, potentially eliciting intense emotional experiences.

Another key theme emerging from this study was social awareness and the strengthening of professional identity. Service-learning contributed to reinforcing the importance of the nursing role, not only from a clinical perspective but also as a health promoter and agent of social change. Students reported feeling more connected to their future professional role. Similar results were found by Henríquez-Melgarejo et al. [35], who observed that the application of service-learning methodology in nursing education was a valuable tool for developing the skills and competencies necessary for effective future professional practice, as well as enhancing interaction with the community. Additionally, participants indicated that this methodology facilitated the development of transversal competencies such as empathy and teamwork. In line with this, Rosen et al. [36] confirmed the positive effects of service-learning as an effective strategy for communication training. Moreover, an international review found that service-learning programs involving active participation of nursing students generate both educational and non-educational benefits, including the development of theoretical and practical learning, communication skills, teamwork, empathy, and social commitment [15]. In terms of its novel contribution, this study addresses an aspect that remains underexplored in the service-learning literature: the connection between professional identity formation and CPR-focused community training. Although the positive impact of service-learning on professional identity has been demonstrated in other nursing contexts (e.g., geriatric care and community health) [10,11], its influence on nursing students’ sense of professional agency in the context of emergency health promotion has received limited attention.

Furthermore, the results indicate that the implementation of service-learning can also be associated with perceived difficulties and barriers, both organizational and personal. Time planning, managing large groups, and coordination were perceived as significant challenges. Specific challenges students may face include fear of being unprepared, anxiety when dealing with difficult participants, or frustration in learning environments. However, these experiences can also serve as opportunities for professional and personal growth. Moreover, educators and institutions should collaborate to overcome structural barriers and provide institutional support for the implementation of service-learning activities.

Finally, it is important to note that in this study, the service-learning methodology was applied specifically to community-based CPR training, an area that remains relatively underexplored in the literature. Within this context, service-learning offers a distinctive strategy for expanding the reach of CPR education through a so-called “multiplier effect,” whereby nursing students not only acquire resuscitation competencies for their future professional practice, but also transmit these skills to community members who might otherwise lack access to formal training. This approach helps to address a key limitation of conventional CPR education, particularly the limited availability of certified instructors and the restricted scalability of traditional classroom-based formats.

This highlights the need to strengthen strategies that enhance this multiplier effect by enabling a greater proportion of citizens to respond effectively in life-threatening emergencies. Nevertheless, the long-term sustainability of this model depends on addressing the organizational challenges identified by students, including difficulties in time planning, coordination of large groups, and the need for stronger institutional support, which are also reflected in the broader service-learning literature. Consequently, the systematic integration of CPR training into service-learning activities appears essential to maximize its potential impact on public health outcomes.

### Limitations

The results of this study should be interpreted in light of several limitations. First, the in-depth interview design involves a degree of subjectivity, and although triangulation strategies and expert review were applied, the influence of social desirability bias cannot be ruled out. The research team also acted as supervisors of the educational intervention within the academic course. This dual role may have influenced participants’ responses, as students might have perceived an implicit expectation to provide positive evaluations of the experience. Therefore, the possibility of social desirability bias cannot be excluded. Although interviews were conducted after the completion of the course and confidentiality was ensured, the academic context may still have affected the openness of participants’ responses and contributed to a more favorable tone in the reported perceptions. Future studies should consider the use of independent interviewers not involved in teaching activities to minimize this potential bias. Another limitation of this study is related to the role of the interviewer in the data collection process. Although the use of field notes on non-verbal communication enriched the contextual understanding of the interviews, the interpretation of these observations may have introduced a degree of subjectivity. Non-verbal cues were recorded and later incorporated into the analytical process, which could have influenced the interpretation of participants’ narratives and contributed to interpretive bias. This potential source of researcher subjectivity should be considered when interpreting the findings. Additionally, the activities were conducted within a specific academic period and context, which may limit the transferability of the findings. Furthermore, although students received formal CPR training and were assessed during the course, no objective pre-assessment of individual CPR skills was conducted immediately prior to the community teaching sessions. This limits our ability to confirm the competency level of each student at the moment of delivering the intervention and should be addressed in future research. Another limitation that should be considered relates to the interpretation of potential differences according to participants’ sociodemographic characteristics. Although some variations in perceptions appeared to emerge depending on factors such as age or previous experience, the qualitative nature of this study, the relatively small sample size, and the limited representation of male participants prevented the establishment of inferential or generalizable conclusions regarding demographic differences. Therefore, these findings should be interpreted cautiously and explored further in future studies with larger and more diverse samples. A significant gap in the existing evidence—and in this study—is the lack of direct comparison between service-learning and other teaching methodologies for CPR training. The absence of a control group or comparative design limits the conclusions that can be drawn about the specific added value of the service-learning approach. Finally, interaction with different educational centers and groups may have introduced variability in the implementation of the learning activities, affecting the consistency of the educational experience.

## 5. Conclusions

The findings of this qualitative study suggest that nursing students perceived service-learning in CPR education as a meaningful educational experience associated with reinforcement of theoretical–practical learning, increased self-confidence, emotional engagement, and greater awareness of the social and educational role of nursing within the community. Participants also identified organizational, emotional, and personal challenges related to the implementation of the activities, highlighting the complexity of applying service-learning methodologies in real educational settings.

This study contributes to the growing body of evidence regarding the potential value of service-learning in nursing education and highlights its possible relevance for community-based CPR training initiatives. Future research incorporating objective competency measures, comparative educational methodologies, and longitudinal follow-up is needed to better understand the educational and practical impact of service-learning on CPR training and professional development.

## Figures and Tables

**Table 1:** Interview guide.

Stage	Subject	Content/Example Questions
Introduction	My intention	I am a member of a research group. Understanding your perceptions could be valuable for exploring experiences related to CPR training through service-learning.
	Ethical issues	Participation in the study is voluntary, and participants may withdraw at any time without providing a reason. The interviews will be audio-recorded to facilitate subsequent data analysis. Under no circumstances will personal information be shared. Prior to starting, signing the informed consent form is required.
Beginning	Introductory question	To begin, could you briefly introduce yourself?/What prior experience did you have with CPR training before participating in the service-learning (SL) teaching innovation project?/What expectations did you have before starting this training using the SL methodology?
Development	Conversation guide	How would you describe, in your own words, the SL methodology?/Which elements of this methodology do you consider had the greatest impact on your learning?/How would you compare SL with other teaching methods you have experienced in your nursing education?/Did you feel supported by the teaching staff?/In what ways do you feel the SL project contributed to improving your practical CPR skills?/Do you think SL helped you better integrate theoretical knowledge with practice? If so, how?/Do you feel more prepared to handle real-life CPR situations after this experience? Why?/Do you feel more capable of teaching CPR after this experience? Why?/How did you feel participating with the community during the SL activities?/Was there any situation during the teaching or practice of CPR in the community that stood out to you?/What were the predominant emotions you recall throughout the process?/In what ways do you think this experience influenced you as a future nursing professional?/Do you think the SL methodology promoted the development of social or communication skills? Which ones?/What difficulties did you face during the implementation of the SL project?/What aspects would you change or improve?
Closing	Final question	To conclude, what lessons or reflections do you take away from this experience? Is there anything else you would like to add?
	Appreciation	Thank you very much for your collaboration; your interview will be extremely valuable for the study.

**Table 2:** Sociodemographic characteristics of the participants.

Participant	Gender	Age	Activity Location
E1	Female	20	School
E2	Female	22	School
E3	Female	20	School
E4	Female	22	School
E5	Male	20	School
E6	Female	22	School
E7	Female	23	School
E8	Female	19	School
E9	Female	20	School
E10	Male	20	Sports Center
E11	Female	28	Sports Center
E12	Female	20	School
E13	Female	22	School
E14	Female	20	School
E15	Female	19	School
E16	Female	20	School
E17	Female	20	School
E18	Female	21	School
E19	Female	22	School
E20	Female	22	School
E21	Female	22	School
E22	Female	27	School
E23	Female	26	School
E24	Female	20	Sports Center
E25	Female	23	School
E26	Female	20	Sports Center
E27	Female	24	Sports Center
E28	Female	48	Cultural Association
E29	Female	23	Sports Center
E30	Female	50	Cultural Association

**Table 3:** Themes, sub-themes and units of meaning.

Themes	Sub-Themes	Units of Meaning
Meaningful Learning and Competency Consolidation	Consolidation of Theoretical and Practical Knowledge	Content Review; deep Understanding; applied Practice.
	Increased security and confidence	Confidence to Act; assurance in Life-Threatening Situations
Emotional Impact Associated with CPR Instruction	Emergence of Positive Emotions	Satisfaction; motivation; enriching experience
	Initially Challenging Emotional Situations	Nervousness; insecurity; pre-Activity Tension
Social Awareness and Strengthening of Professional Identity	Recognition of the Professional Role as a Community Agent	responsibility; vocation; professional vision
	Perception of Social Impact and the Importance of Health Education	Applicability; connection with society.
Perceived Difficulties and Barriers in the Implementation of Service-learning	Structural and Organizational Limitations of the Activity	Limited time; large groups; coordination
	Individual Difficulties Perceived by Students	Shame; fear of making mistakes

## Data Availability

The data presented in this study are available on request from the corresponding author due to ethical reasons.

## Public Involvement Statement

No public involvement in any aspect of this research.

## Guidelines and Standards Statement

This manuscript was drafted against the guidelines of the Consolidated Criteria for Reporting Qualitative Research (COREQ).
